# Economic Impacts and Quality of Life for Caregivers of Patients with Retinitis Pigmentosa: A Cross-Sectional Japanese Study

**DOI:** 10.3390/healthcare11070988

**Published:** 2023-03-30

**Authors:** Katsuhiko Watanabe, Yoshimune Hiratsuka, Shuichi Yamamoto, Akira Murakami

**Affiliations:** 1 Medical Affairs Division, Novartis Pharma K.K., Tokyo 105-6333, Japan; 2 Department of Ophthalmology, Juntendo University Graduate School of Medicine, Tokyo 113-8421, Japan; 3 Japan Community Health Care Organization, Tokyo 108-8583, Japan

**Keywords:** economic impact, caregivers, Japan, quality of life, retinitis pigmentosa

## Abstract

Retinitis pigmentosa (RP) is the second leading cause of visual impairment in Japan and causes progressive vision loss in affected patients. Caregiving for patients with RP is associated with socioeconomic impacts; however, data on the magnitude and scope of these impacts are lacking. This cross-sectional study surveyed informal caregivers of patients with RP in Japan. The questionnaire assessed the socioeconomic status of participants; work impacts through the Work Productivity and Activity Impairment Questionnaire adapted for caregivers; and quality of life impacts through the Japanese version of the Caregiver Reaction Assessment (CRA) and the 5-level EQ-5D version (EQ-5D-5L). Of the 37 participating caregivers, 28 (75.7%) were employed. Among those, the average annual income was 2,722,080 yen (*n* = 20) and the mean loss of work productivity was 6.6%. The mean EQ-5D-5L index score was 0.882, and the mean CRA total score was 2.1. A mild to very severe impact on family life, leisure and hobbies, social life, and mental health was experienced by 83.8%, 78.4%, 75.7%, and 70.3%, respectively. These results suggest that caregivers of patients with RP may be disadvantaged in terms of employment and income and may experience wide-ranging impacts on their quality of daily life.

## 1. Introduction

Retinitis pigmentosa (RP) is a group of hereditary eye disorders with over 60 implicated mutations [1]. Patients with RP experience a progressive loss of rod and cone cells in the retina, with the symptoms of visual impairment first presenting as loss of night vision and narrowing of the visual field [1]. In severe cases, the continued loss of vision can result in complete blindness [1]. Although treatments have been developed for many eye diseases and the proportion of patients who develop visual impairment has been decreasing globally [2], there are currently no disease-modifying therapies that can completely reverse or halt the progression of RP [1].

In Japan, RP is the second leading cause of visual impairment [3]. Although supportive care is available for affected patients, the disease has considerable socioeconomic impacts for patients [4]. However, questions remain regarding the magnitude and scope of the impacts experienced by caregivers of patients with RP, both financially and in terms of their overall quality of daily life and wellbeing. Although caregivers of patients with RP in Japan have access to some support, including financial aid and leave for caregiving duties [5,6,7,8], less support is available for caregivers than for patients. We must first understand the impacts on caregivers in order to identify areas for additional support. Although it is reported that RP is associated with a high level of caregiver burden arising from psychological and financial stress [9], there have been few detailed investigations of the burdens experienced by caregivers of patients with RP thus far.

This study aimed to investigate the socioeconomic and quality of life (QOL) burden experienced by informal caregivers (including family, relatives, and friends) of patients with RP in Japan.

## 2. Materials and Methods

### 2.1. Study Design

This cross-sectional study surveyed informal caregivers of patients with RP in Japan between September 2021 and November 2021. Participants were surveyed using a quantitative web-based questionnaire; the full questionnaire is provided as Data S1. The questionnaire was originally developed for this study and was supervised by board members of the Japanese Retinitis Pigmentosa Society (JRPS) and three ophthalmologists specialized in retinal disorders. A preliminary test was also conducted with two caregivers to confirm the understandability and usability of the web-based questionnaire. The questionnaire included assessments of the socioeconomic status of participants, as well as the Work Productivity and Activity Impairment Questionnaire (WPAI:GH) adapted for caregivers [10,11,12], the Japanese version of the Caregiver Reaction Assessment (CRA) [13], and the 5-level EQ-5D version (EQ-5D-5L) [14]. Where necessary, permissions were obtained to reproduce these questionnaires.

The study was conducted in accordance with the ethical principles that have their origin in the Declaration of Helsinki and complied with Novartis regulatory standards and all Japanese legal and regulatory requirements. All study materials (i.e., the study protocol, questionnaire, and informed consent form) were approved by the Ethics Committee of the Medical Corporation Fujikeikai Kitamachi Clinic prior to data collection. All study participants provided informed consent and were provided with information about the purpose of the study.

### 2.2. Participants

The study participants were informal caregivers of patients with a diagnosis of RP who have or had a Specific Medical Expenses Recipient Certificate (for Designated Intractable Diseases) for RP, regardless of gene variance status identification, and who were aged 18–99 years. Caregivers of patients with Usher syndrome, Bardet–Biedl syndrome, mucopolysaccharidosis, Kearns–Sayre syndrome, adult Refsum disease, infantile Refsum disease, Alagille syndrome, Bassen–Kornzweig syndrome, Cockayne syndrome, Hallervorden–Spatz syndrome, or Rud syndrome were excluded from the study. Participants were recruited via the JRPS. The request for participation in the survey was made by the JRPS through an announcement on a member-specific website, which was also distributed to members via e-mail. Participants could then voluntarily access the web-based questionnaire from the link provided in the announcement.

### 2.3. Socioeconomic Outcomes

The following socioeconomic outcomes (including employment, productivity, and financial aid) were assessed: the percentage of caregivers who were employed, students, and not employed (including those who had retired); the occupational classification of caregivers; the productivity and activity impacts of caregiving, as assessed using the WPAI:GH adapted for caregivers [12]; the annual income of caregivers; and the presence of financial aid received by caregivers.

The WPAI:GH was created as a patient-reported quantitative assessment of the amount of absenteeism, presenteeism, and daily activity impairment attributable to general health. Outcomes are expressed as impairment percentages, with higher numbers indicating greater impairment and less productivity, i.e., worse outcomes [10]. The construct validity has been tested for use in clinical trials, along with its reproducibility [10]. The Japanese translation of WPAI:GH used in this study was created by a professional translation service, using independent translations, harmonization, back-translation, expert review, and review by local language users [11].

Confounding factors included caregiver age, sex, occupation, relationship with the patient, and the patient’s degree of visual impairment. To minimize the effect of confounding factors, the age and sex groups were matched when comparing the socioeconomic status of participants with the general population.

### 2.4. QOL Outcomes

The QOL outcomes of caregivers (including emotional and psychological measures) were assessed using the CRA [13] and the EQ-5D-5L [14]. 

The CRA is a multidimensional scale that has been used in a large number of studies for caregivers of cancer patients. It consists of five subscales: caregiver’s self-esteem, impact on schedule, lack of family support, impact on health, and impact on finances. Each item is answered using a five-point Likert scale with responses ranging from 1 (strongly agree) to 5 (strongly disagree). The composite scores are computed as averages of the items within each subscale, ranging from 1.0 to 5.0. Higher scores on the negative dimensions represent higher levels of perceived burden. Therefore, the higher the score, the higher the perceived burden. The Japanese version of the CRA has been shown to have sufficient reliability and validity [13].

The EQ-5D is the most widely used multi-attribute utility instrument for measuring health-related QOL in cost-effectiveness analyses. The EQ-5D-5L descriptive system comprises five dimensions: mobility, self-care, usual activities, pain/discomfort, and anxiety/depression. Each dimension has five levels: no problems, slight problems, moderate problems, severe problems, and extreme problems. The level selected for each dimension results in a single-digit number. The digits for the five dimensions can be combined into a 5-digit number that describes the respondent’s health state. The EQ-5D index value is derived by applying a formula that attaches values (weights) to each of the levels in each dimension. It is calculated by deducting the appropriate weights from 1, the value for full health (i.e., state 11111), and a calculation method of the EQ-5D index value specifically for Japanese respondents has been previously developed [15,16]. The EQ visual analogue scale (EQ VAS) records the respondent’s self-rated health on a vertical visual analogue scale, where the endpoints are labelled “The best health you can imagine” (100) and “The worst health you can imagine” (0). The VAS can be used as a quantitative measure of a health outcome that reflects the respondent’s own judgement [14].

### 2.5. Statistical Analyses

Summary statistics (mean, median, confidence interval, and standard deviation) were calculated for each assessed variable. Missing data to specific questions, including responses of “Prefer not to say” or “Not sure”, were excluded from the summary statistics analysis. Questionnaire responses were aggregated using Excel (Microsoft Corp., Redmond, WA, USA) and BellCurve Hideyoshi Dplus v 1.10 (Social Survey Research Information Co., Ltd., Tokyo, Japan), and statistically analyzed using BellCurve for Excel (add-in software on Excel; Social Survey Research Information Co., Ltd., Tokyo, Japan).

## 3. Results

### 3.1. Participants

The full analysis set included a total of 37 caregivers of patients with RP; the demographics of the survey participants are shown in Table 1. The mean age of caregivers was 54.5 years (*n* = 34), and the majority of caregivers were female (25/37; 67.6%). In total, 27/37 caregivers were aged 18–64 years (73.0%) and 7/37 caregivers were aged ≥65 years (18.9%). The majority of participants were spouses of patients with RP (20/37; 54.1%). Moreover, 12/37 participants were parents or guardians of children with RP (32.4%), and 2/37 participants were children of adults with RP (5.4%).

### 3.2. Outcome: Socioeconomic Impacts (Employment, Productivity, and Financial Aid)

The employment status of caregivers is shown in Table 1. Employed caregivers included those who were self-employed, family workers, or employed by companies or corporations. The overall employment rate of caregivers was 75.7% (28/37). Additionally, 17 female and 9 male participants reported being employed by a company or corporation, with a mean age of 53.3 years for the female participants (*n* = 16) and 53.8 years for the male participants (*n* = 8). Among these participants, 17.6% (3/17) of the female participants and 77.8% (7/9) of the male participants stated that they were in regular employment (including full-time and part-time permanent employment). The occupations of the employed participants were categorized according to the Standard Occupational Classification of Japan [17] as follows: professional and engineering workers, 9/28 (32.1%); clerical workers, 4/28 (14.3%); sales workers, 4/28 (14.3%); service workers, 4/28 (14.3%); administrative and managerial workers, 1/28 (3.6%); construction and mining workers, 1/28 (3.6%); and unclassified, 5/28 (17.9%).

The WPAI scores of caregivers are shown in Table 2. In caregivers who were employed (*n* = 28), the overall work productivity impairment due to patient health problems was 6.6%, and the activity impairment due to patient health problems was 16.8%. 

The income of caregivers is shown in Table 3. Overall, the average annual income of caregivers who were employed was 2,722,080 yen (*n* = 20). Female and male caregivers who were employed had a mean annual income of 1,570,018 yen (*n* = 12, mean age: 51.9 years) and 4,450,173 yen (*n* = 8, mean age: 54.0 years), respectively. Only 2/32 participants (5.4%) reported receiving financial aid for being a caregiver of a patient with RP.

### 3.3. Outcome: QOL Impacts

The QOL impacts on caregivers are shown in Table 4. The mean EQ-5D-5L index value was 0.882, and the mean CRA total score was 2.1. Out of the 37 respondents, 11 (29.7%) reported experiencing depression, 20 (54.1%) experienced anxiety, 19 (51.4%) experienced frustration, and 8 (21.6%) experienced guilt; 10/37 (27.0%) reported not experiencing any of these emotional and psychological impacts.

**Figure 1:**
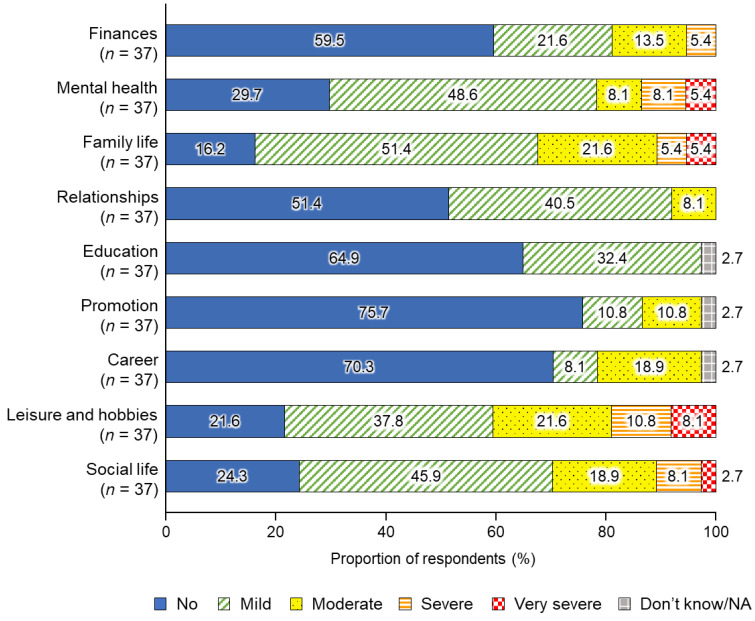
Self-reported overall impact of caregiving on the lives of caregivers of patients with retinitis pigmentosa. NA, not applicable.

The self-reported overall impact of caregiving on caregivers’ lives is shown in Figure 1. For family life, leisure and hobbies, social life, and mental health, the majority of caregivers (83.8%, 78.4%, 75.7%, and 70.3%, respectively) responded that care had a mild to very severe influence on their life. The percentage of respondents who answered that the impact of care is severe or very severe was 10.8% for family life, 18.9% for leisure and hobbies, 10.8% for social life, and 13.5% for mental health. Conversely, for promotion, career, education, finance, and relationships, the majority (75.7%, 70.3%, 64.9%, 59.5%, and 51.4%, respectively) responded that care had no influence at all.

The CRA score of each subscale is shown in Table S1. The mean value was 1.9 for impact on caregiver schedules, 3.1 for caregiver self-esteem, 1.6 for lack of family support, 1.6 for impact on health, and 1.5 for impact on finances.

## 4. Discussion

Although it is reported that RP is associated with a high level of caregiver burden arising from psychological and financial stress [9], this is the first study that reports in detail the subjective burden of caregivers of patients with RP in Japan. Our findings illustrate that caregivers may be disadvantaged both socioeconomically (in terms of their career and remuneration) and in terms of their daily life quality and wellbeing.

The loss of work productivity due to caring for patients with RP was 6.6%; although there have been no published reports using the WPAI for caregivers of patients with other ophthalmological diseases, this loss is lower than has been previously reported for other diseases. For example, for caregivers of patients with chronic disease [18] and caregivers of patients with an osteoporotic fracture [19], loss of work productivity has previously been reported to be 40.7% and 61.4%, respectively. However, patients with osteoporotic fracture were older (mean age: 78 years) and more likely to be female [19], which may have contributed to the higher loss of work productivity experienced by their caregivers. Furthermore, this discrepancy may reflect the lack of established treatment for RP, and, therefore, fewer caregiving activities associated with treatment (e.g., taking patients to hospital appointments, organizing medical appointments, and supporting patients with their medication). In addition, patients with visual impairment may be more likely to receive community or institutional support (e.g., guide dogs, learning aids, support for leading an independent life, and specialist nursing homes for patients with visual impairments), which may alleviate some of the demands required of the caregiver.

Nonetheless, the caregivers of patients with RP in our study had lower employment rates overall compared with the equivalent sex- and age-matched groups in Japan [20]. The employment rate of caregivers was 72.0% for women (mean age: 54.2 years) and 83.3% for men (mean age: 55.1 years), which is lower than the general population employment rates reported for women aged 45–54 years (78.7%) and men aged 55–59 years (91.0%) in Japan in 2021 [20]. The mean annual incomes of caregivers in employment were 1,570,018 yen for women (mean age: 51.9 years) and 4,450,173 yen for men (mean age: 54.0 years); these incomes are much lower than the estimated annual income in Japan for women aged 50–54 years (4,234,800 yen) and men aged 50–54 years (6,848,600 yen) [21]. Furthermore, nearly half (40.5%) of the surveyed participants responded that being a caregiver of a person with visual impairment had an impact on their finances. Thus, the perceived financial impacts and objective economic burden experienced by caregivers of patients with RP are generally consistent. Additionally, as there is currently no effective therapy to prevent the progression of RP [1], caregivers who are currently employed may need to reduce their working hours as the symptoms of the patient for whom they are caring worsen. Together, these findings suggest that caregivers of RP patients may be disadvantaged in terms of both employment and income. A similar employment disadvantage has been reported for caregivers of adults with mental illness, other cognitive/behavioral conditions, and physical conditions with or without secondary mental illness [22].

We used questionnaires, including the EQ-5D-5L and the CRA, to investigate the subjective burden and QOL of caregivers of patients with RP. The caregivers reported wide-ranging impacts on their daily life, including family life, leisure and hobbies, social life, and mental health. In addition, some caregivers responded that the impact of caregiving on these aspects of life was severe or very severe. Conversely, the CRA scores indicated that dissatisfaction with family support and impact on general health status were not severe. The CRA scores can be compared with caregivers of patients with other conditions: caregivers of patients with RP experience a lower burden than caregivers of patients with neovascular age-related macular degeneration [23], rheumatoid arthritis [24], dementia [25], colorectal cancer [26], multiple sclerosis [27], and atrial fibrillation [28]. In addition, the mean score of the EQ-5D-5L (0.882) was lower than the age-matched score in the overall Japanese population (50–59 years, male: 0.936; female: 0.928) [29]. Although there are no published reports of EQ-5D-5L scores of caregivers of patients with other ophthalmological diseases, the mean score of caregivers in our study was similar to the previously reported scores of caregivers of patients with cancer (0.73) [30], dementia (0.885) [31], and Alzheimer’s disease/dementia (0.79) [32] in Japan. Previously reported risk factors for increased caregiver burden include female sex, low education, living with the patient, having a higher number of caregiving hours, having a lack of choice in being a caregiver, and experiencing depression, social isolation, or financial stress [33]. We did not find a clear connection between specific caregiver demographics and an increased risk of caregiver impacts; however, this is likely because of the limited sample size in our study.

We acknowledge the limitations of this study. Only members of the JRPS or related individuals were included, which may have resulted in bias pertaining to participant background. Furthermore, as this study is based on the self-reported experience of the caregivers, the results may be affected by recall bias. In addition, we observed a higher proportion of individuals in professional and engineering occupations (32.1%) than reported in the general population in Japan in 2021 (18.8%); although, the distribution of other occupations was similar to the published proportions [20]. It is unclear if the discrepancy in professional and technical employment observed in this study reflects the actual occupations of caregivers of RP patients in Japan, or whether there is another underlying explanation (for example, caregivers in these professions were more likely to have the time and inclination to respond to a survey). Larger-scale exploration of caregiver characteristics would be needed to determine whether there are meaningful implications or biases related to this finding.

## 5. Conclusions

This study is the first to comprehensively examine the burden experienced by caregivers of patients with RP in Japan. The socioeconomic burden of caregivers was surveyed, and the results indicated that caregivers of patients with RP may be disadvantaged in terms of employment and income. Additionally, the results suggest that caring for patients with RP has a wide range of impacts on the QOL and psychological wellbeing of caregivers.

## Figures and Tables

**Table 1:** Demographics of survey participants.

	Participants *N* = 37
Age (*n* = 34)	
Mean (SD)	54.5 (14.0)
Median (min, max)	57.0 (29, 84)
95% CI	[49.6, 59.4]
Q1, Q3	41.3, 63.0
Age group, years, *n* (%)	
18 to 64	27 (73.0)
>64	7 (18.9)
Unknown	3 (8.1)
Sex, *n* (%)	
Female	25 (67.6)
Male	12 (32.4)
Employment, *n* (%)	
Working	
*Student*	0 (0.0)
*Non-student*	28 (75.7)
Non-working	
*Student*	1 (2.7)
*Non-student*	8 (21.6)
Relationship to patient, *n* (%)	
Parent or guardian	12 (32.4)
Spouse	20 (54.1)
Child	2 (5.4)
Relative	1 (2.7)
Friend	1 (2.7)
Other	1 (2.7)
Age group of patients, years, *n* (%)	
<18	0 (0.0)
18 to 64	28 (75.7)
>64	9 (24.3)

CI, confidence interval; Q, quartile; SD, standard deviation.

**Table 2:** Work productivity and activity impacts on caregivers of patients with retinitis pigmentosa.

	*n* (%)	Percent Work Time Missed Due to Patient’s Health, Mean (SD)	Percent Impairment While Working Due to Patient’s Health, Mean (SD)	Percent Overall Work Impairment Due to Patient’s Health, Mean (SD)	Percent Activity Impairment Due to Patient’s Health, Mean (SD)
Overall	28 (100.0)	0.6 (2.7)	6.1 (12.0)	6.6 (12.4)	16.8 (20.7)
Age group, years					
18 to 64	23 (82.1)	0.8 (3.0)	3.9 (7.2)	4.6 (8.3)	14.3 (17.8)
>64	3 (10.7)	0.0 (0.0)	16.7 (28.9)	16.7 (28.9)	23.3 (25.2)
Unknown	2 (7.1)	0.0 (0.0)	15.0 (21.2)	15.0 (21.2)	35.0 (49.5)
Sex					
Female	18 (64.3)	0.8 (3.4)	2.2 (4.3)	2.9 (6.3)	12.2 (15.9)
Male	10 (35.7)	0.3 (1.0)	13.0 (17.7)	13.3 (17.6)	25.0 (26.4)
Employment					
Regular	10 (35.7)	0.3 (1.0)	8.0 (12.3)	8.3 (12.4)	20.0 (25.8)
Non-regular	16 (57.1)	0.9 (3.6)	5.0 (12.6)	5.8 (13.4)	12.5 (14.8)
Self-employed	2 (7.1)	0.0 (0.0)	5.0 (7.1)	5.0 (7.1)	35.0 (35.4)
Relationship to patient					
Parent or guardian	11 (39.3)	1.3 (4.3)	0.9 (3.0)	2.1 (6.9)	8.2 (10.8)
Spouse	14 (50.0)	0.2 (0.9)	10.7 (15.4)	10.9 (15.4)	22.9 (22.7)
Child	1 (3.6)	0.0 (-)	10.0 (-)	10.0 (-)	60.0 (-)
Relative	1 (3.6)	0.0 (-)	0.0 (-)	0.0 (-)	0.0 (-)
Friend	0 (0.0)	-	-	-	-
Other	1 (3.6)	0.0 (-)	0.0 (-)	0.0 (-)	0.0 (-)
Age group of patients, years					
<18	0 (0.0)	-	-	-	-
18 to 64	25 (89.3)	0.1 (0.6)	6.4 (12.5)	6.5 (12.6)	17.2 (21.5)
>64	3 (10.7)	4.8 (8.2)	3.3 (5.8)	7.6 (13.2)	13.3 (15.3)

SD, standard deviation.

**Table 3:** The annual income of caregivers of patients with retinitis pigmentosa.

	*n*	Age, Mean (SD)	Annual Income (Yen/Year)			
			Mean (SD)	Median (Min, Max)	95% CI	Q1, Q3
Overall	20	52.8 (15.3)	2,722,080 (3,099,572)	1,000,000 (220, 10,000,000)	[1,271,436, 4,172,724]	500,000, 5,000,000
Female	12	51.9 (15.6)	1,570,018 (1,907,738)	820,000 (220, 6,000,000)	[357,899, 2,782,137]	500,000, 1,250,000
Male	8	54.0 (15.9)	4,450,173 (3,830,109)	3,700,000 (360, 10,000,000)	[1,248,122, 7,652,223]	1,850,255, 7,200,000

CI, confidence interval; Q, quartile; SD, standard deviation.

**Table 4:** Quality of life impacts on caregivers of patients with retinitis pigmentosa.

	*n* (%)	CRA Score, Mean (SD)	EQ-5D-5L, Mean (SD)		Emotional and Psychological Experience, *n* (%)				Taking Medication for Depression or Anxiety, *n* (%)
		Total	Index	VAS	Depression	Anxiety	Frustration	Guilt	
Overall	37 (100.0)	2.1 (0.6)	0.882 (0.116)	77.8 (12.2)	11 (29.7)	20 (54.1)	19 (51.4)	8 (21.6)	0 (0.0)
Age group, years									
18 to 64	27 (100.0)	2.1 (0.7)	0.881 (0.114)	77.4 (13.4)	8 (29.6)	14 (51.9)	15 (55.6)	6 (22.2)	0 (0.0)
>64	7 (100.0)	2.2 (0.3)	0.897 (0.131)	80.1 (8.2)	2 (28.6)	4 (57.1)	3 (42.9)	1 (14.3)	0 (0.0)
Unknown	3 (100.0)	2.3 (0.9)	0.851 (0.134)	76.7 (11.5)	1 (33.3)	2 (66.7)	1 (33.3)	1 (33.3)	0 (0.0)
Sex									
Female	25 (100.0)	2.1 (0.6)	0.892 (0.110)	77.0 (13.4)	7 (28.0)	13 (52.0)	13 (52.0)	5 (20.0)	0 (0.0)
Male	12 (100.0)	2.1 (0.6)	0.861 (0.129)	79.6 (9.6)	4 (33.3)	7 (58.3)	6 (50.0)	3 (25.0)	0 (0.0)
Employment									
Working									
*Student*	0 (100.0)	-	-	-	-	-	-	-	-
*Non-student*	28 (100.0)	2.1 (0.7)	0.881 (0.109)	78.7 (12.2)	10 (35.7)	16 (57.1)	15 (53.6)	8 (28.6)	0 (0.0)
Non-working									
*Student*	1 (100.0)	2.2 (-)	1.000 (-)	70.0 (-)	0 (0.0)	1 (100.0)	1 (100.0)	0 (0.0)	0 (0.0)
*Non-student*	8 (100.0)	2.2 (0.6)	0.870 (0.143)	75.8 (13.4)	1 (12.5)	3 (37.5)	3 (37.5)	0 (0.0)	0 (0.0)
Relationship to patient									
Parent or guardian	12 (100.0)	1.9 (0.6)	0.911 (0.115)	80.1 (13.9)	4 (33.3)	7 (58.3)	4 (33.3)	5 (41.7)	0 (0.0)
Spouse	20 (100.0)	2.3 (0.6)	0.862 (0.121)	76.1 (12.3)	7 (35.0)	11 (55.0)	13 (65.0)	3 (15.0)	0 (0.0)
Child	2 (100.0)	2.2 (0.0)	0.907 (0.132)	72.5 (3.5)	0 (0.0)	2 (100.0)	2 (100.0)	0 (0.0)	0 (0.0)
Relative	1 (100.0)	1.5 (-)	0.814 (-)	78.0 (-)	0 (0.0)	0 (0.0)	0 (0.0)	0 (0.0)	0 (0.0)
Friend	1 (100.0)	1.6 (-)	1.000 (-)	85.0 (-)	0 (0.0)	0 (0.0)	0 (0.0)	0 (0.0)	0 (0.0)
Others	1 (100.0)	1.2 (-)	0.829 (-)	90.0 (-)	0 (0.0)	0 (0.0)	0 (0.0)	0 (0.0)	0 (0.0)
Age group of patients, years									
<18	0 (100.0)	-	-	-	-	-	-		-
18 to 64	28 (100.0)	2.2 (0.7)	0.860 (0.113)	77.1 (13.2)	10 (35.7)	17 (60.7)	16 (57.1)	8 (28.6)	0 (0.0)
>64	9 (100.0)	1.9 (0.5)	0.949 (0.103)	80.1 (8.8)	1 (11.1)	3 (33.3)	3 (33.3)	0 (0.0)	0 (0.0)

CRA, Caregiver Reaction Assessment; EQ-5D-5L, The 5-level EQ-5D version; SD, standard deviation; VAS, visual analogue scale.

## Data Availability

The datasets generated during and/or analyzed during the current study are available from the corresponding author on reasonable request.

## Supplementary Materials

The following supporting information can be downloaded at https://www.mdpi.com/article/10.3390/healthcare11070988/s1, Data S1: Questionnaire; Table S1: Caregiver Reaction Assessment scores of caregivers of patients with retinitis pigmentosa.
