# Supplementary material for: Economic Impacts and Quality of Life for Caregivers of Patients with Retinitis Pigmentosa: A Cross-Sectional Japanese Study
Source: Healthcare (Basel). 2023 Mar 30;11(7):988. doi: 10.3390/healthcare11070988 (PMC10093847; doi:10.3390/healthcare11070988)
Supplement: Supplementary file 1 [file healthcare-11-00988-s001.zip › healthcare-2145508-supplementary.pdf]

Economic Impacts and Quality of Life for Caregivers of Patients with Retinitis Pigmentosa: A Cross-Sectional Japanese Study

Katsuhiko Watanabe [1,*], Yoshimune Hiratsuka [2], Shuichi Yamamoto [3], Akira Murakami [2]

[1]Medical Affairs Division, Novartis Pharma K.K., Tokyo, Japan

[2]Department of Ophthalmology, Juntendo University Graduate School of Medicine, Tokyo, Japan

[3]Japan Community Health Care Organization, Tokyo 108-8583, Japan

Corresponding author:

Katsuhiko Watanabe

Toranomon Hills Mori Tower 23-1, Toranomon 1-chome, Minato-ku, Tokyo 105-6333, Japan

Phone: +81 80-3026-4262

Email: katsuhiko.watanabe@novartis.com

ORCID iD: 0000-0002-3060-0926

Supplementary materials.

Supplementary Data S1. Questionnaire.

Module 1 About You (the Caregiver for a Patient with Retinitis Pigmentosa or LCA)

Q1. Are you;

a. Female

b. Male

c. Prefer not to say

Q2. What is your age?

a. years

b. Prefer not to say

Q3. Do you have any physical problem (subjective symptom) such as illness or wound in these few days?

a. Yes

b. No

c. Prefer not to say

Q4. What is the age of your care-receiver?

a. Less than 18 years

b. 18 to less than 65 years

c. 65 years or older

Q5. Which of the following best describes your relationship with your care-receiver?

a. Parent or guardian

b. Spouse

c. Child

d. Other relatives

e. Friend

f. Other – please specify

Q6. Which of the following best describes your situation? Select **all** that apply.

a. I am in full-time work.

b. I work part-time.

c. I have given up work to provide care for the patient with retinitis pigmentosa or LCA.

d. I have reduced my working hours to provide care for the patient with retinitis pigmentosa or LCA.

e. I receive government financial aid to provide care for the patient with retinitis pigmentosa or LCA.

f. Other – please specify.

Module 2 Work

The following questions will ask you in detail about different aspects of your employment and how your care-receiver's health problems may have impacted your work. Also, in order to learn about your usual situation properly, you will be asked in detail how the spread of COVID-19 infections impacted your current situation.

The following questions ask you about the effect of your care-receiver's health problems on your work and regular activities. The health problems here mean any physical or mental problem or symptom. *Please select an answer as instructed or enter a checkmark.*

Q1. Are you currently employed (working for pay)?_____ No ____Yes

The next questions are about a **past seven-day period**, not including today.

Q2. During the past seven days, how many hours did you miss from work because of <u>your care-receiver's health problems</u>? *Include hours you missed on sick days, times you went in late, left early, etc., because of your care-receiver's health problems. Do not include time you missed to participate in this survey. (If 0 hour, please enter "0" [zero].)*

________hour(s)

Q3. During the past seven days, how many hours did you miss from work because of any reason other than your care-receiver's health problems, such as vacation, holidays, time off to participate in this survey? (If 0 hour, please enter "0" [zero].)

________hour(s)

Q4. During the past seven days, how many hours did you actually work? (If 0 hour, please enter the number "0.")

________hour(s)

Q5. During the past seven days, how much did your care-receiver's health problems affect your productivity <u>while you were working</u>?

Think about days you were limited in the amount or kind of work you could do, days you accomplished less than you would like, or days you could not do your work as carefully as usual. If your care-receiver's health problems affected your work only a little, choose a low number. Choose a high number if they affected your work a great deal.

Consider only how much <u>your care-receiver's health problems</u> affected your productivity <u>while you were working</u>.

○ ○ ○ ○ ○ ○ ○ ○ ○ ○ ○

Your care-receiver's health problems had no effect on work	0	1	2	3	4	5	6	7	8	9	10	Your care-receiver's health problems completely prevented you from working

Q6. During the past seven days, how much did your care-receiver's health problems affect your regular daily activities, other than work at a job?

The regular activities mean the usual activities you do, such as work around the house, shopping, childcare, exercising, studying, etc. Think about times you were limited in the amount or kind of activities you could do and times you accomplished less than you would like. If your care-receiver's health problems affected your activities only a little, choose a low number. Choose a high number if they affected your activities a great deal.

Consider only how much <u>your care-receiver's health problems</u> affected your regular daily activities, other than work at a job.

Your care-receiver's health problems had no effect on my daily activities	○ 0	○ 1	○ 2	○ 3	○ 4	○ 5	○ 6	○ 7	○ 8	○ 9	○ 10	Your care-receiver's health problems completely prevented you from doing your daily activities

Q7. During the past seven days, how many hours has the spread of COVID-19 infections impacted the time you missed from work because of your care-receiver's health problems? (If 0 hour, please enter "0" [zero].)

Answer: hour(s)

Q8. How many hours has the spread of COVID-19 infections impacted the time you missed from work because of any reason **<u>other</u>** than your care-receiver's health problems? (If 0 hour, please enter "0" [zero].)

Answer: hour(s)

Q9. **How many hours** do you **usually** work per week?
Answer: hour(s)

Q10. During the **last one year,** how many full and partial days (e.g. 1.5 days) did you miss from work because of any other reason including refreshment and illness? (If 0 day, please enter “0” [zero].)

Answer: day(s)

Q11. During the last one year, how many full and partial days (e.g. 1.5 days) did you miss from work due to your care-receiver’s visual impairment? (If 0 day, please enter “0” [zero].)

Answer: day(s)

Q12. How does your care-receiver’s visual impairment impact your job performance over **a regular four-week period**? Select from the following 6-scale from 0 to 5.

0. No impact	1. Mild impact	2. Mild to moderate impact	3. Moderate impact	4. Moderate to significant impact	5. Significant impact
○	○	○	○	○	○

Q13. Which of the following best describes your current state of work?

a. I work as a full-time worker

b. I regularly work as a part-time worker

c. Self-employed

d. Casual work (Not working on a regular basis)

e. Student (Working part-time)

f. Other – please specify

Q14. Which of the following best describes your current state of work?

a. Student (Not working)

b. Vocational training (Not working)

c. Retired

d. Not employed (looking for work)

e. Not employed (not looking for work but working around the house, childcare, providing care, etc.)

f. Unable to work

g. Other – please specify

Q15. How many years have you worked at your current place of employment?

Answer: year(s)

Q16. Please describe your current occupation.

Answer: Complete freely.

Q17. What was your annual income you earned from working in the last year (total annual income before tax and insurance fee)?

a. ____ yen
b. Prefer not to say

Q18. Has the spread of COVID-19 infections impacted your work or employment status?

a. Yes
b. No

Q19. Does your care-receiver work at present?

a. Yes
b. No

Q20. Please select any articles for daily use that your care-receiver currently uses to support his/her vision in his/her working environment. Select all that apply.

a. Computers (only select if specialized for him/her)
b. Magnifiers
c. Magnification apparatus for reading
d. Screen reader, spoken word processor, or verbal calculator
e. Braille display, braille books, braille device
f. Modifications to mobile phones such as applications (apps)
g. Digital voice recorder (voice reading machine)
h. Not using any items (exclusion)
i. Other – please specify:

Q21. How much money do you spend on average per year for articles for daily use to support your care-receiver's vision in his/her working environment? (If 0 yen, please enter "0" [zero]. If you cannot answer, select "Cannot answer.")

a. yen ○ Cannot answer

Q22. Has the spread of COVID-19 infections changed how much money you spend on articles for daily use to support your care-receiver's vision in his/her working environment?

a. Increased
b. Decreased
c. Unchanged

Q23. How much more money do you spend? (If you cannot answer, select “Cannot answer.”)

Amount of money you spend more: yen ○ Cannot answer

Q24. How much less money do you spend? (If you cannot answer, select “Cannot answer.”)

Amount of money you spend less: yen ○ Cannot answer

Q25. Please select the most appropriate reason for your care-receiver not currently using articles for daily use to support his/her vision in his/her working environment.

a. He/she does not require articles for daily use to support his/her vision
b. He/she cannot use articles for daily use to support his/her vision
c. Other – please specify:

Module 3 Financial Aid

The following questions will ask you in detail about financial aid you may receive for being a caregiver for an individual with a visual impairment.

Q1. Do you receive any financial aid such as a public donation (charity), public benefit, funds from friends and relatives because you are a caregiver to an individual with a visual impairment?

a. Yes
b. No
c. Unsure

Q2. If you receive any financial aid because you are a caregiver to an individual with a visual impairment, which of the following forms of assistance do you receive? Select all that apply. Also, how much money do you receive per month?

a. Public donation (charity): yen
b. Public benefit: yen
c. Funds from friends and relatives: yen
d. Other – please specify: yen
e. Prefer not to say

Module 4 Your Wellbeing

Q1. What impact does your role as a caregiver for an individual with a visual impairment have on the following areas of your life? (Enter one checkmark each in a box in the horizontal line.)

	No impact	Mild impact	Moderate impact	Severe impact	Very severe impact	Don’t know/NA
	0	1	2	3	4	5
Social life	○	○	○	○	○	○
Leisure and hobbies	○	○	○	○	○	○
Career	○	○	○	○	○	○
Career progression	○	○	○	○	○	○
Education	○	○	○	○	○	○
Relationships	○	○	○	○	○	○
Family life	○	○	○	○	○	○
Mental health	○	○	○	○	○	○
Finances	○	○	○	○	○	○

Q2. Have you ever felt depressed due to your caring requirements for an individual with a visual impairment?

a. Yes

b. No

Q3. In what situation have you felt depressed? Please describe in concrete terms.

- Prefer not to say
- Column for providing the answer:

Q4. Have you ever felt anxious due to your caring requirements for an individual with a visual impairment?

a. Yes

b. No

Q5. In what situation have you felt anxious? Please describe in concrete terms.

- Prefer not to say
- Column for providing the answer:

Q6. Are you taking medication to manage feelings of depression or anxiety due to your caring requirements for an individual with a visual impairment?

a. Yes

b. No

Q7. Have you ever experienced feelings of frustration or guilt due to your caring requirements for an individual with a visual impairment? Select all that apply.

a. No

b. I have experienced frustration

c. I have experienced guilt

d. Prefer not to say

Q8. In what situation have you experienced feelings of frustration or guilt? Please describe in concrete terms.

• Prefer not to say

• Column for providing the answer:

Q9. You are asked about what you are feeling about providing care.
Enter a checkmark for only one number that applies. (Enter one checkmark each for a number in the horizontal line.)

	Do not think so at all	Do not think so	Neither of these	Think so	Always think so
Impact on daily living					
1. Opportunities to visit my relatives or friends decreased after starting to provide care.	1○	2○	3○	4○	5○
2. My current main activity is caregiving.	1○	2○	3○	4○	5○
3. My schedule or plan decreased after starting to provide care.	1○	2○	3○	4○	5○
4. I have to stop work or another activity in the middle of it for giving care.	1○	2○	3○	4○	5○
5. I do not have time to relax myself due to caregiving.	1○	2○	3○	4○	5○
Acceptance of care					

1. I am proud of being able to give care to my family member.	1○	2○	3○	4○	5○
2. Caregiving is important for me.	1○	2○	3○	4○	5○
3. I initiatively want to give care.	1○	2○	3○	4○	5○
4. To give care makes me happy.	1○	2○	3○	4○	5○
5. I am enjoying giving care.	1○	2○	3○	4○	5○
	Do not think so at all	Do not think so	Neither of these	Think so	Always think so
Family's support					
1. My other family members (siblings, children) rely solely on me to care for the patient.	1○	2○	3○	4○	5○
2. Everyone forces only me to take care of the patient.	1○	2○	3○	4○	5○
3. I feel like being abandoned by other family members after starting caregiving.	1○	2○	3○	4○	5○
4. It is very difficult to have support from other family members for giving care.	1○	2○	3○	4○	5○
Impact on your health condition					
1. My health condition has got worse after starting caregiving.	1○	2○	3○	4○	5○
2. I am always tired after starting caregiving.	1○	2○	3○	4○	5○
Financial impact					
1. I am forced to bear financial burden due to caregiving.	1○	2○	3○	4○	5○
2. It is difficult to pay costs for caregiving.	1○	2○	3○	4○	5○
	Do not think so at all	Do not think so	Neither of these	Think so	Always think so

Module 5 About Your Today's Health Condition

From here, you will be asked about your today's health condition and how you think about health.

The 5-level EQ-5D version (Questions are copyrighted and not listed.)

Supplementary Table S1. Caregiver Reaction Assessment scores of caregivers of patients with retinitis pigmentosa.

	n (%)	CRA score, mean (SD)					
		**Impact on schedule**	**Caregiver self-esteem**	**Lack of family support**	**Impact on health**	**Impact on finances**	**Total**
Overall	37 (100.0)	1.9 (1.0)	3.1 (1.1)	1.6 (0.8)	1.6 (0.8)	1.5 (0.9)	2.1 (0.6)
Age group, years							
18 to 64	27 (73.0)	1.9 (1.0)	3.0 (1.1)	1.6 (0.8)	1.6 (0.8)	1.5 (0.9)	2.1 (0.7)
>64	7 (18.9)	1.9 (0.8)	3.5 (1.1)	1.5 (0.5)	1.4 (0.8)	1.5 (0.6)	2.2 (0.3)
Unknown	3 (8.1)	2.1 (1.5)	3.1 (1.3)	2.0 (1.1)	1.8 (1.4)	1.7 (1.2)	2.3 (0.9)
Sex							
Female	25 (67.6)	1.9 (1.0)	3.3 (1.2)	1.6 (0.8)	1.5 (0.8)	1.4 (0.8)	2.1 (0.7)
Male	12 (32.4)	2.1 (0.9)	2.7 (0.8)	1.8 (0.7)	1.6 (0.9)	1.8 (1.0)	2.1 (0.6)
Employment							
Working							
Student	0 (0.0)	—	—	—	—	—	—
Non-student	28 (75.7)	1.9 (1.0)	3.0 (1.2)	1.7 (0.9)	1.6 (0.8)	1.5 (0.9)	2.1 (0.7)
Non-working							
Student	1 (2.7)	1.4 (-)	4.6 (-)	1.3 (-)	1.0 (-)	1.0 (-)	2.2 (-)
Non-student	8 (21.6)	2.1 (0.9)	3.3 (1.0)	1.6 (0.7)	1.6 (0.9)	1.6 (0.9)	2.2 (0.6)
Relationship to patient							
Parent or guardian	12 (32.4)	1.9 (1.0)	2.5 (1.1)	1.6 (0.6)	1.4 (0.5)	1.6 (1.0)	1.9 (0.6)
Spouse	20 (54.1)	2.2 (1.0)	3.4 (1.0)	1.8 (1.0)	1.8 (1.0)	1.6 (0.9)	2.3 (0.6)

Child	2 (5.4)	1.3 (0.1)	4.7 (0.1)	1.3 (0.0)	1.3 (0.4)	1.0 (0.0)	2.2 (0.0)
Relative	1 (2.7)	1.6 (-)	2.2 (-)	1.0 (-)	1.0 (-)	1.0 (-)	1.5 (-)
Friend	1 (2.7)	1.0 (-)	3.0 (-)	1.0 (-)	1.0 (-)	1.0 (-)	1.6 (-)
Other	1 (2.7)	1.0 (-)	1.8 (-)	1.0 (-)	1.0 (-)	1.0 (-)	1.2 (-)
Age group of patients, years							
<18	0 (0.0)	—	—	—	—	—	—
18 to 64	28 (75.7)	2.0 (1.0)	3.1 (1.1)	1.7 (0.9)	1.6 (0.8)	1.6 (1.0)	2.2 (0.7)
>64	9 (24.3)	1.8 (1.0)	3.0 (1.2)	1.3 (0.4)	1.4 (0.7)	1.2 (0.4)	1.9 (0.5)

CRA, Caregiver Reaction Assessment; SD, standard deviation.
